# Identification of epitopes recognised by mucosal CD4^+^ T-cell populations from cattle experimentally colonised with *Escherichia coli* O157:H7

**DOI:** 10.1186/s13567-016-0374-5

**Published:** 2016-09-02

**Authors:** Alexander Corbishley, Timothy K. Connelley, Eliza B. Wolfson, Keith Ballingall, Amy E. Beckett, David L. Gally, Tom N. McNeilly

**Affiliations:** 1 Farm Animal Practice, Royal (Dick) School of Veterinary Studies, The University of Edinburgh, Easter Bush, Midlothian, EH25 9RG UK; 2 Division of Immunity and Infection, The Roslin Institute, The University of Edinburgh, Easter Bush, Midlothian, EH25 9RG UK; 3 Department of Pathology, University of Cambridge, Tennis Court Road, Cambridge, CB2 1QP UK; 4 Moredun Research Institute, Pentlands Science Park, Bush Loan, Penicuik, EH26 0PZ UK

## Abstract

Vaccines targeting enterohaemorrhagic *Escherichia coli* (EHEC) O157:H7 shedding in cattle are only partially protective. The correlates of protection of these vaccines are unknown, but it is probable that they reduce bacterial adherence at the mucosal surface via the induction of blocking antibodies. Recent studies have indicated a role for cellular immunity in cattle during colonisation, providing an impetus to understand the bacterial epitopes recognised during this response. This study mapped the epitopes of 16 EHEC O157:H7 proteins recognised by rectal lymph node CD4^+^ T-cells from calves colonised with Shiga toxin producing EHEC O157:H7 strains. 20 CD4^+^ T-cell epitopes specific to *E. coli* from 7 of the proteins were identified. The highly conserved N-terminal region of Intimin, including the signal peptide, was consistently recognised by mucosal CD4^+^ T-cell populations from multiple animals of different major histocompatibility complex class II haplotypes. These T-cell epitopes are missing from many Intimin constructs used in published vaccine trials, but are relatively conserved across a range of EHEC serotypes, offering the potential to develop cross protective vaccines. Antibodies recognising H7 flagellin have been consistently identified in colonised calves; however CD4^+^ T-cell epitopes from H7 flagellin were not identified in this study, suggesting that H7 flagellin may act as a T-cell independent antigen. This is the first time that the epitopes recognised by CD4^+^ T-cells following colonisation with an attaching and effacing pathogen have been characterised in any species. The findings have implications for the design of antigens used in the next generation of EHEC O157:H7 vaccines.

**Electronic supplementary material:**

The online version of this article (doi:10.1186/s13567-016-0374-5) contains supplementary material, which is available to authorized users.

## Introduction

Enterohaemorrhagic *Escherichia coli* (EHEC) O157:H7 causes diarrhoea and potentially fatal renal failure in humans as a result of Shiga toxin (Stx) activity [1]. Cattle are the primary reservoir for EHEC O157:H7 [2] and this has led to the development of two licensed vaccines aimed at reducing EHEC O157 carriage in cattle. Econiche (Econiche Corp, Belleville, Canada), is manufactured from culture supernatants containing type III secreted proteins (T3SPs), whilst the other is manufactured from cell membrane extracts (Epitopix, Willmar, USA) prepared from iron restricted cultures. Both vaccines are only partially protective [3] and the correlates of protection have not yet been determined.

The primary site of colonisation in cattle is the terminal rectum where the bacteria form attaching and effacing (A/E) lesions on the apical epithelial surface [4]. We have recently shown that CD4^+^ T-cells infiltrate the rectal mucosa during experimental colonisation of cattle with EHEC O157:H7 and that CD4^+^ T-cells isolated from the rectal lymph nodes of colonised calves proliferate in response to T3SPs [5]. Furthermore, transcriptional profiling of the rectal mucosa during colonisation reveals a bias towards a T-helper type 1 (T_H_1) response, with colonisation inducing increased levels of interferon-gamma (IFN-γ) and *T*-*bet* transcripts within rectal mucosal tissues [5]. This suggests that cellular immunity may play an important role in controlling EHEC O157:H7 in cattle, particularly as cattle clear EHEC O157:H7 despite only generating low and highly variable mucosal antibody titres to key bacterial antigens [6]. Thus we hypothesise that while antibody production following vaccination may block binding to the epithelium, as suggested by passive immunisation studies [7], vaccines that induce a cellular response may be more effective in clearance once bacteria have formed A/E lesions with epithelial cells.

Given the importance of CD4^+^ T-cells in coordinating humoral and cellular immunity, an understanding of the epitopes that drive this response is essential to the design of protective vaccines, specifically with respect to establishing a CD4^+^ T-cell memory pool that can respond to epitopes presented during natural colonisation. Additionally, as CD4^+^ T-cells recognise linear epitopes presented on major histocompatibility complex (MHC) Class II molecules, studying the epitope repertoire recognised following colonisation allows basic bioinformatics tools to be used to compare these epitopes to the sequences of other EHEC serotypes of public health concern (O26, O111, O103, O121, O45 and O145). Epitopes that are conserved between these serotypes may provide cross-protection between EHEC serotypes.

The aim of this study was to characterise the epitopes recognised by mucosal CD4^+^ T-cells following experimental colonisation with Stx producing EHEC O157:H7. To achieve this, we measured CD4^+^ T-cell responses to overlapping peptides from 16 EHEC proteins that have previously been shown to play a role in colonisation or immunity to EHEC O157:H7 in cattle and/or have been previously used in experimental EHEC vaccines.

## Materials and methods

**Figure 1:**
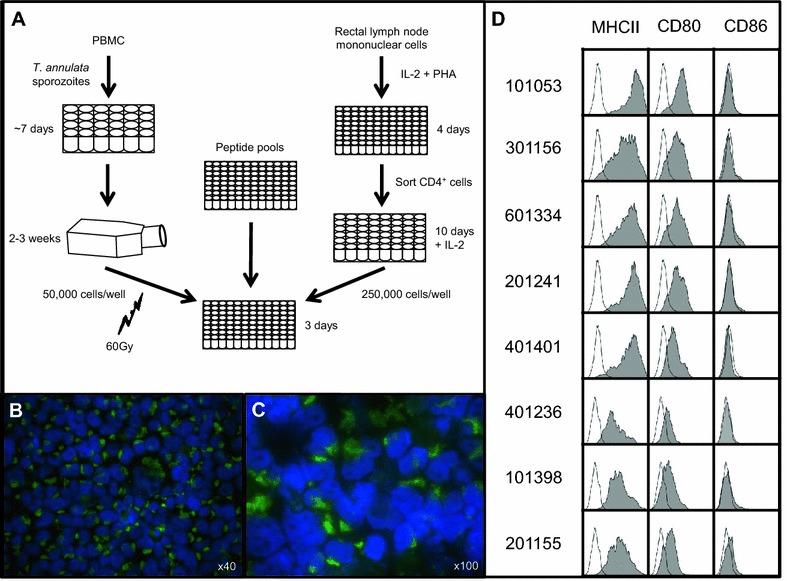
**Experimental approach. A** Graphical representation of APC and CD4^+^ T-cell line generation. **B**, **C** Immunofluorescent staining of *T. annulata* immortalised PBMC. Nuclei stained using DAPI (blue) and macroschizonts stained using monoclonal antibody 1C12 (green). **D** Flow cytometric analysis following staining of *T. annulata* immortalised PBMC for MHCII, CD80 and CD86. Filled histograms denote stained samples, open histograms denote the no primary control.

The experimental approach is summarised in Figure 1A. CD4^+^ T-cells from the rectal lymph node (RLN) of experimentally colonised calves were expanded using an approach adapted from [8]. Autologous APCs were generated by immortalisation of peripheral blood mononuclear cells (PBMC) following *Theileria annulata* infection. These cells express MHCII and efficiently present exogenous antigen to CD4^+^ T-cells [9]. Cryopreserved PBMC and RLN cells were available from eight calves experimentally colonised with EHEC O157:H7 in a previous study [5], which was performed at the Moredun Research Institute (MRI) in accordance with the UK. Animals (Scientific Procedures) Act, 1986 under Home Office license 60/3179. The study was approved by the MRI Animal Experiments and Ethical Review Committee. All cultures were at 37 °C in a humidified 5% CO_2_ atmosphere.

### Peptide library

The length of peptides presented by MHCII in cattle has not been defined. In humans, the optimum MHCII binding affinity occurs with peptides 18–20 amino acids in length [10]. Lyophilised N-terminus amine, C-terminus glycine acid 18mer peptides overlapping by 12 amino acids were supplied by JPT Peptide Technologies (Berlin, Germany) for 16 bacterial proteins (see Additional file 1). 330 µM stock solutions were prepared in 5% dimethyl sulfoxide (DMSO) in phosphate buffered saline (PBS). Sequential peptides from each protein were pooled in groups of 10 (details in Additional file 2). *Theileria parva* peptides (Additional file 3) were prepared as a negative control to the same final concentration in 5% DMSO in PBS.

### APC line generation

1 × 10^7^ PBMC were resuscitated and infected with *Theileria annulata* sporozoites in lymphocyte culture medium (LCM) containing: Roswell Park Memorial Institute (RPMI) 1640 medium (Gibco), 10% heat inactivated (HI) fetal calf serum (FCS), 200 mM l-glutamine, 100 IU/mL penicillin and 100 µg/mL streptomycin (Invitrogen). Cultures were incubated in 24 well plates and inspected daily. Once growing rapidly, cells were transferred to flasks. APC lines were considered established after 2 weeks of culture.

### Flow cytometric analysis of APC lines

Cells were incubated with anti-bovine CD80 (ILA159 [11]), CD86 (ILA190 [11]) or MHCII (CC158 [12]) antibodies, washed and incubated with goat anti-mouse IgG fluorescein isothiocyanate (FITC) conjugated antibody (Molecular Probes, Paisley, UK); unstained and no primary antibody controls were included. Incubations were for 30 min at 4 °C and 5% FCS/0.02% sodium azide/PBS was used for washes and antibody dilutions. Cells were analysed using a FACS Calibur flow cytometer (BD Biosciences, San Jose, USA) and FlowJo v10.0.7 (FlowJo, Ashland, USA).

### Immunofluorescent microscopic assessment of APC lines

Cells were pelleted on to glass slides using the Cytospin system (Thermo Scientific, Paisley, UK) and fixed in acetone. Slides were stained using the Sequenza Immunostaining Center (Thermo Scientific) using 50 µL reaction volumes with reagents diluted in PBS as follows: cells were incubated with anti-*T. annulata* p104 vb (1C12 [13]) followed by goat anti-mouse IgG FITC conjugated antibody (Molecular Probes) then 3 nM DAPI. Slides were mounted using Fluorescent Mounting Media (Agilent Technologies, Wokingham, UK) and examined using a DMLB Fluorescent Microscope (Leica, Wetzlar, Germany).

### CD4^+^ T-cell line generation

1 × 10^7^ RLN cells were resuscitated into LCM medium containing 1 µg/mL phytohaemagglutinin (PHA) (Thermo Scientific) and 100 IU/mL rhIL-2 (Novartis, Basel, Switzerland) and seeded into round bottomed 96 well plates at 35 000 cells/well. After 4 days, wells were pooled and cells stained using anti-bovine CD4 AlexaFluor 647 conjugated antibody (CC8, AbD Serotec, Oxford, UK) diluted in 0.5% bovine serum albumin (BSA) (Sigma) in PBS. CD4^+^ cells were sorted at 4 °C using an Aria IIIu FACS machine (BD Biosciences). Cells in LCM containing 100 IU/mL rhIL-2 were seeded into 48 well plates at 1 × 10^6^ cells/well, incubated for 10 days.

### Peptide stimulations

Fourteen days after PHA stimulation, 250 000 CD4^+^ T-cells/well in LCM were seeded into 96 well plates and 50 000 γ-irradiated (60 Gy) APCs in LCM added to each well. Test and control peptide pools were added to a final concentration of 3 µM per peptide; approximately 1 µg/well (average MW 1932 Da). One µg/mL PHA was used as a positive control. Plates were incubated for 3 days. Interferon-γ (IFN-γ) release into the supernatant was measured using a commercial bovine IFN-γ ELISA kit (Mabtech AB, Nacka Strand, Sweden) and a microplate reader (Tecan, Männedorf, Switzerland). Individual peptides from up to eight positive pools were then tested individually. The process was as per the pools, except that peptide TC4 (Additional file 3) was used as a negative control and the final concentration of DMSO was 0.05 versus 0.5% for the pools.

### MHC II genotyping of calves

DNA was extracted from the APC lines using the DNeasy Blood and Tissue DNA extraction kit (Qiagen, Hilden, Germany). Exon 2 of the bovine MHC Class II *DRB3* gene was amplified using GoTaq DNA Polymerase (Promega, Southampton, UK) as per the manufacturer’s instructions. The forward (F455: TATCCCGTCTCTGCAGCACATTTC) and reverse (R329: CACCCCCGCGCTCACCTCGCCGC) primers were a modification of those previously published for genotyping the ovine *DRB1* locus [14]. All reactions yielded a single amplicon of the predicted size (301 bp) which was purified and sequenced. Alleles were assigned as detailed in [14].

### Peptide alignments

Peptide alignments were performed using the translated BLAST (tblastn, National Center for Biotechnology Information (NCBI)) and exported for manual inspection in Excel v14.0.7128 (Microsoft, Seattle, USA). Where necessary, annotations were obtained for accession numbers using Batch Entrez (NCBI). R package seqinr was used to append annotations to tblastn query results. Intimin alignments were performed using Clustal Omega and formatted using MView (EBI).

### Data analysis

R v3.1.0 and packages ggplot2, GGally, scales, grid and gridExtra was used for analysis and plots. IFN-γ release in response to non-reactive peptides was assumed to be normally distributed around the mean IFN-γ release from the negative control wells, with reactive peptides skewing the overall distribution of the data. The standard deviation was therefore calculated manually where the mean was the IFN-γ release from the negative control wells and the deviance from this mean calculated using the wells where IFN-γ release was less than this mean. Positive wells were defined as wells where IFN-γ release was >3 standard deviations from the mean of the negative control wells.

## Results

### APC cell line phenotypes

Autologous *Theileria annulata* infected cell lines were established for all eight animals to be utilised as APC. Subsets of lines were stained to confirm *T. annulata* infection (Figures 1B and C). All eight lines exhibited MHCII and CD80 expression (Figure 1D). CD86 expression was undetectable.

### Peptide pool screening

Twelve proteins were chosen because they had been shown to be targets of the antibody response in cattle colonised with EHEC O157:H7 (Intimin, Tir, EspA, EspB, EspD, EspM2, NleA, TccP, FliC, FliD and Stx2a and Sxt2c B-subunits [15–18]) and would therefore be expected to contain CD4^+^ T-cell epitopes. A further four secreted effector proteins were selected (EspF, EspK, NleC and NleD) as our previous studies suggested that T3SP preparations containing increased secreted effector proteins but minimal translocon-associated proteins stimulate stronger lymphoproliferative responses in rectal lymph node cells compared to T3SP preparations predominantly consisting of translocon-associated proteins [5]. IFN-γ release was used as a readout as this study also demonstrated the presence of T_H_1 responses in colonised cattle, and rectal lymph node cells released IFN-γ following re-stimulation with EHEC O157 T3SPs.

The CD4^+^ T-cell line from animal 101 053 exhibited high background IFN-γ release (mean of 30 439 pg/mL in the negative control wells compared to 120 326 pg/mL in PHA controls; ~25% of the PHA positive control) and the CD4^+^ T-cell expansion for animal 101 398 was less efficient, yielding only enough cells to seed 110 000 cells/well. Consequently the assay lacked sensitivity and specificity respectively for these animals (Additional file 4) and they were excluded from subsequent analysis. Mean IFN-γ release in response to PHA by the lines from the other 6 animals ranged from 14 070 pg/mL to 42 682 pg/mL. Background IFN-γ release by the negative control wells ranged from 1.9 to 7.5% of the PHA positive control.

**Figure 2:**
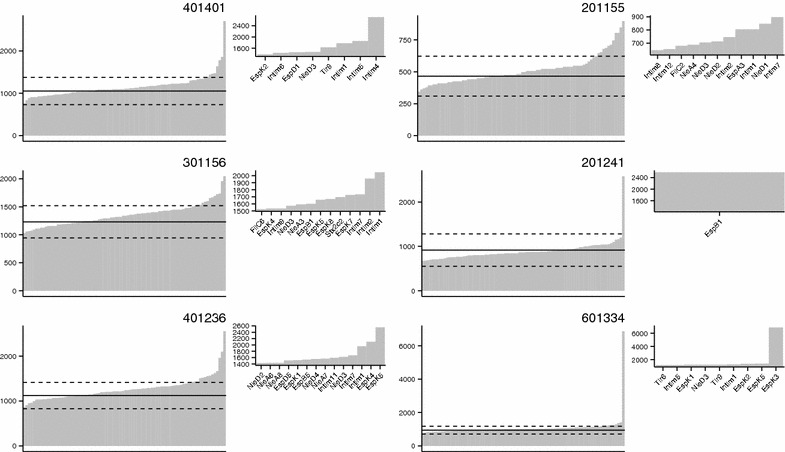
**IFN-γ production by rectal CD4** ^**+**^ **T-cells of EHEC O157 colonised calves in response to pooled bacterial peptides.** CD4^+^ T-cell lines prepared from the rectal lymph nodes of animals challenged with EHEC O157:H7 were stimulated with pools of peptides from 16 EHEC O157:H7 proteins and subsequent IFN-γ release quantified by ELISA. Each panel represents results from a single animal. Peptide pools are ordered on the Y axis by the amount of IFN-γ produced. Peptide pool names are given for positive pools only. Solid lines denote the mean of two negative control wells. Dotted lines denote three standard deviations from the negative control as detailed in the "Materials and methods" section. Positive pools are shown in the inset next to each graph.

IFN-γ release by each cell line in response to the 84 peptide pools is shown in Figure 2, with positive pools inset next to each graph. Positive pools are also summarised for each animal in Table 1 alongside the *DRB3* MHCII haplotypes. None of the animals were of the same haplotype, demonstrating the outbred nature of the calves. Thirty-six positive pools were identified, representing 10/16 proteins tested. Ten of these pools were from Intimin, seven from EspK and five from NleD. Lines from 5/6 animals demonstrated responsiveness to at least one Intimin and NleD pool and 4/6 to at least one EspK pool.

**Table 1:** **Peptide pools eliciting an increase in IFN-γ production by rectal CD4** ^**+**^ **T-cells from EHEC O157:H7 colonised calves**

	Calf 401401	Calf 201155	Calf 301156	Calf 201241	Calf 401236	Calf 601334
*DRB3* haplotype	DRB3*2703 DRB3*1501	DRB3*1001 DRB3*1801	DRB3*1001 DRB3*1201	DRB3*0101 DRB3*0701	DRB3*0101 DRB3*1101	DRB3*14011 DRB3*1101
*Peptide pool*						
EspA3		X				
EspB1			X	X		
EspB5					X	
EspD1	X					
EspD5					X	
EspK1					X	X
EspK2	X					X
EspK3						X
EspK4			X		X	
EspK5			X		X	X
EspK7			X			
EspK8			X			
FliC2		X				
FliC6			X			
Intim1	X	X	X		X	X
Intim2		X	X			
Intim4	X					
Intim5	X					X
Intim6	X					
Intim7		X	X		X	
Intim8		X				
Intim9			X			
Intim11					X	
Intim12		X				
NleA3			X			
NleA4		X				
NleA6					X	
NleA7					X	
NleA8					X	
NleD1		X				
NleD2		X			X	
NleD3	X	X	X		X	X
NleD4					X	
Stx2c2			X			
Tir6						X
Tir9	X					X

### Peptide pool deconvolution

**Figure 3:**
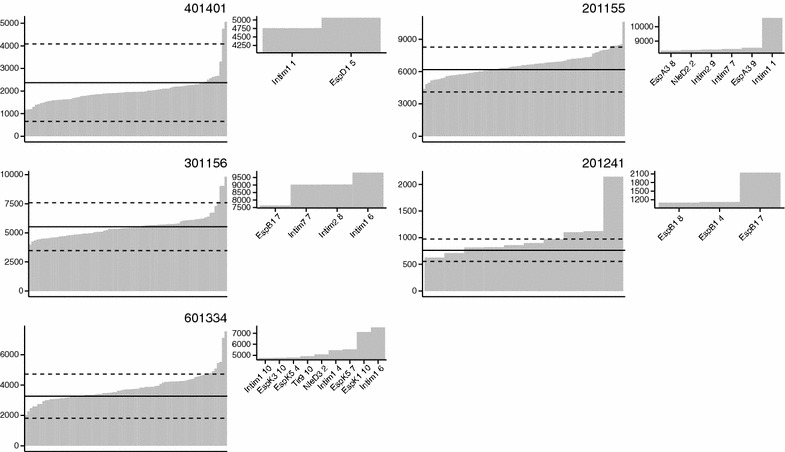
**IFN-γ production by rectal CD4** ^**+**^ **T-cells of EHEC O157 colonised calves in response to individual bacterial peptides.** CD4^+^ T-cell lines prepared from the rectal lymph nodes of animals challenged with EHEC O157:H7 were stimulated with individual peptides from the top eight positive pools for each animal in Table 1 and subsequent IFN-γ release quantified by ELISA. Each panel represents results from a single animal. Peptides are ordered on the Y axis by the amount of IFN-γ produced. Peptide names are given for positive peptides only. Solid lines denote the mean of four negative control wells. Dotted lines denote three standard deviations from the negative control as detailed in the "Materials and methods" section. Positive peptides are shown in the inset next to each graph.

**Figure 4:**
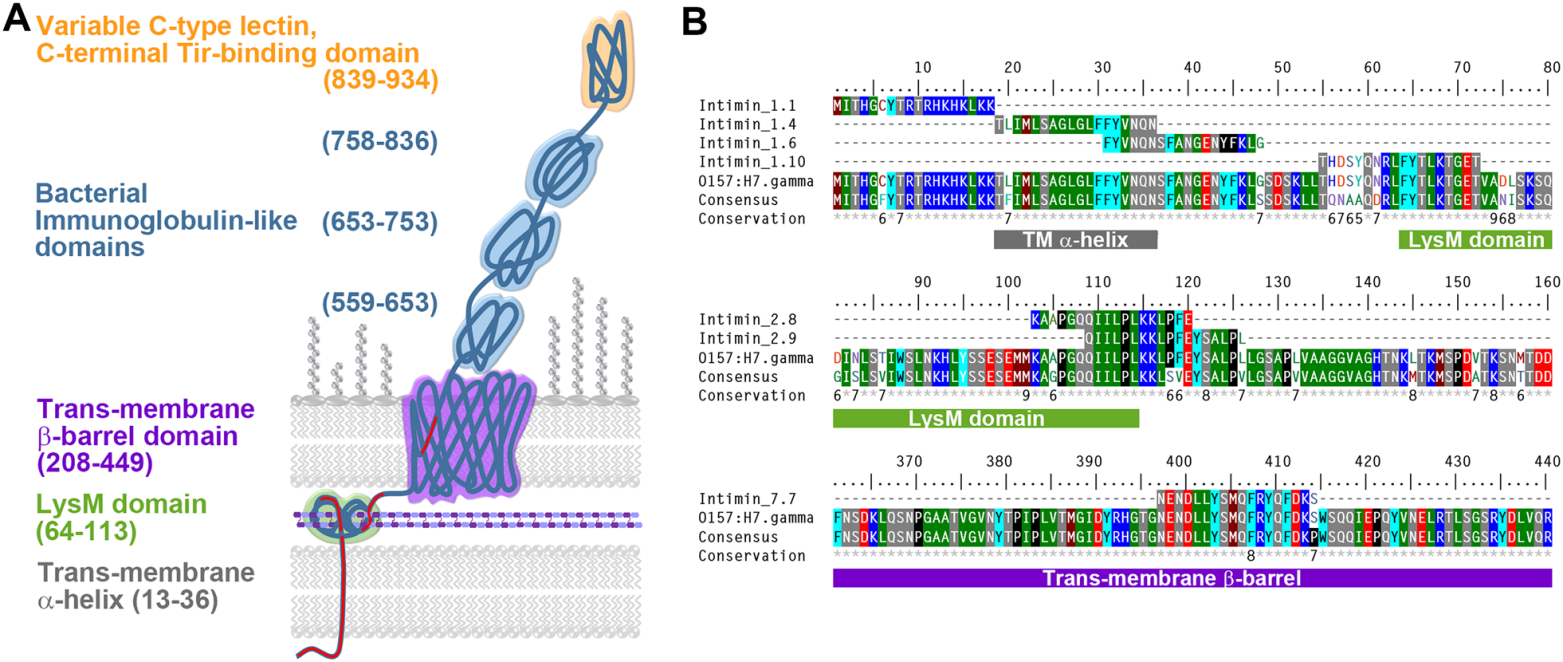
**Location and conservation of bovine CD4** ^**+**^ **T-cell Intimin epitopes after experimental colonisation with EHEC O157:H7. A** Schematic of EHEC Intimin-γ, labelled with structural domain coordinates (Uniprot accession: P43261). Location of CD4^+^ T-cell epitopes is indicated in red. **B** Sequence alignment of CD4^+^ T-cell epitopes against the parental EHEC O157:H7 Intimin-γ sequence (accession BAB37982.1) and an Intimin consensus sequence. Conservation of this consensus is indicated on a scale of (*)-0, where * = 100% identity, 9 is highly conserved, down to 0, which is not conserved. Structural domains shown in A are also highlighted in this alignment. The consensus sequence was generated by a MUSCLE [32] multiple sequence alignment of representative Intimin sequences from EHEC of public health concern: O157:H7 (γ, accession BAB37982.1); O145:H28 (γ, accession AHY73165.1); O26:H11 (β, accession BAI28422.1); O103:H2 (ε, accession BAI32332.1); O121:H19 (ε, accession KDV53260.1); O45:H2 (ε, accession EZA22403.1); O111:H- (τ, accession BAI37528.1) and a prototypical EPEC O127:H6 comparator (α, accession YP_002331401.1). Intimin types are indicated in brackets. Conservation levels of this consensus sequence were generated using the PRALINE consistency output [33].

Where sufficient CD4^+^ T-cells were available, individual peptides were assessed from up to eight positive pools. Overall IFN-γ release was higher, likely due to the reduced DMSO concentration in each well. IFN-γ release in response to PHA ranged from 31 360 to 525 433 pg/mL. Negative control wells ranged from 0.1 to 10.8% of the PHA positive control. IFN-γ release in response to the individually deconvolved peptides is shown in Figure 3. From a total of five calves, 20 unique positive peptides were identified. Intimin was the most commonly recognised protein, with 7/20 positive peptides representing sequences from the highly conserved N-terminal region of Intimin (Figure 4).

A tblastn alignment of the amino acid sequence for each peptide was performed using the nr/nt database and 1714 hits with 100% coverage and 100% identity selected. After excluding synthetic sequences, the only alignments not attributed to *E. coli* were hits for Intimin from *E. albertii* and *Shigella boydii* and NleD and EspK from *E. albertii*.

To investigate the degree of conservation between EHEC serotypes, a tblastn alignment for each peptide using the 16 complete EHEC genomes was performed. The percent identity for each peptide is summarised in Table 2. Peptide identity was 100% across the six O157:H7 and four O145:H28 genomes, with the exception of the NleD peptides, where no alignments were returned for the O145:H28 genomes. NleD is encoded by cryptic prophage CP-933 K and a tblastn alignment of the Sakai protein sequence (accession NP308877.1) against the O145:H28, O103:H2, O111:H- and O26:H11 genomes yielded no hits. There were no hits for peptides EspD1.5 and Tir9.10 against the O103:H2, O111:H- and O26:H11 genomes. Tblastn alignments of the EspD and Tir Sakai protein sequences (accessions BAB37978.1 and BAB37984.1 respectively) against the genomes of these three serotypes resulted in single hits of 72–74% and 60–66% identity respectively, indicating a lower level of homology for these proteins. The alignments for the remaining peptides against the whole genome sequences of the O103:H2, O111:H- and O26:H11 strains varied from 72 to 100% identity. As anticipated, no hits were identified for any of the peptides in the three O104:H4 genomes, which do not express a T3SS [19].

**Table 2:**
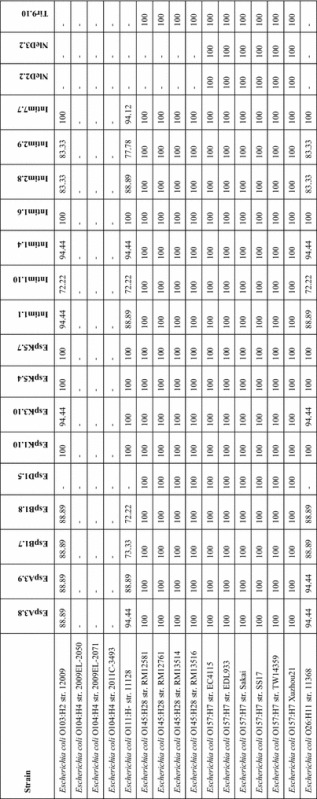
**Conservation of EHEC O157 CD4** ^**+**^ **T-cell epitope sequences across non-O157 EHEC strains**

Maximum percent identity returned by a tblastn query for each reactive peptide from Figure 3 against the complete genome sequences of 16 non-O157 EHEC strains.

– Denotes no hit.

### Intimin alignments

As the most commonly recognised epitopes in colonised cattle were from the N-terminal region of Intimin, these sequences were aligned with the β, γ, ε and θ Intimin sequences from the six other major EHEC serotypes (O145:H28, O26:H1, O103:H2, O121:H19, O45:H2 and O111:H-) and α Intimin from enteropathogenic *E. coli* (EPEC) O127:H6 using Intimin from EHEC O157:H7 as a scaffold (Additional file 5). All 7 Intimin peptides originate from a region of the molecule that is highly conserved between EHEC serotypes. A tblastn alignment of Sakai Intimin (accession BAB37982.1) against all deposited O157:H7 sequences (excluding Sakai), identified 11/12 sequences with 100% identity and 1/12 sequences with 99% identity. The same alignment for Intimin from the O145:H28 strain RM12581 (accession AHY73165.1) for all deposited O145:H28 sequences (excluding RM12581) resulted in 3/3 sequences with 100% identity.

## Discussion

A rational screening approach was undertaken to investigate CD4^+^ T-cell epitopes recognised by cattle following EHEC O157:H7 colonisation. *T. annulata* transformed PBMC were used as APCs. These lines have been shown to efficiently process and present peptides in vitro [9], although potential impacts of *T. annulata* transformation on antigen processing and presentation cannot be excluded. Positive peptides were not identified from all of the positive peptide pools identified in the initial screening experiment, probably as the frequency of peptide-specific CD4^+^ T-cells within these cell lines was too low for detection. Epitopes were identified from EspA, EspB, EspD, EspK, Intimin, NleD and Tir. Serological responses have been detected to all of these proteins with the exception of EspK and NleD. As these are injected effector proteins, it is possible that these epitopes are more efficient at driving cellular rather than humoral responses.

All six animals from which CD4 epitope data were analysed in this study demonstrated rising anti-H7 flagellin IgA and/or IgG1 serum titres during colonisation (data not shown) and H7 flagellin is known to induce strong antibody responses in colonised calves [20, 21]. The failure to identify a convincing CD4^+^ T-cell response against either FliC or FliD peptides suggest that anti-flagellin antibody production during *E. coli* O157 colonisation may largely occur independently of T-cell help as flagellins are known to activate B cells in a T-cell independent manner [22].

Intimin epitopes were identified in 5/6 animals, with all the peptides originating from the intracellular N-terminal region of the molecule, over regions that are identical between sequenced strains of the same serotype and highly conserved between different serotypes. Intimin contains an N-terminal signal peptide, which in EPEC has historically been thought to be cleaved following translocation of the protein into the periplasm [23]. Experimental evidence of signal peptide cleavage in either EPEC or EHEC has not been published, whilst recent models for the biogenesis and secretion of Intimin do not propose cleavage of the signal peptide [24]. The peptides identified in this study sit predominantly within the signal peptide and are adjacent to a LysM domain (Figure 4) which is thought to enable peptidoglycan tethering in the periplasmic space [25]. The Intimin1.6 peptide identified in this study spans the putative signal peptide cleavage site between residues 39 and 40. If the Intimin signal peptide is cleaved, this would suggest that antigen presenting cells in vivo are processing cytoplasmic Intimin.

Intimin is 934 amino acids long. Due to its size and technical challenges associated with expressing full-length Intimin, C-terminal Intimin fragments of varying sizes have been used in vaccine studies. The longest C_899_ [7] and shortest C_280_ [26] fragments have been used in studies of passive transfer of immunity in pigs and cattle respectively where memory recall responses were not relevant. C_531_ [27] and C_280_ [28] fragments have been trialled in cattle and a C_280_ [29] fragment has also been trialled in goats as part of a fusion protein. These shorter Intimin fragments excluded all the epitopes identified in this study. This study suggests that the N-terminus of Intimin is an important immunological target and whilst C-terminal fragments may result in protective antibody titres, the use of full length Intimin or shorter constructs that include the N-terminal amino acid sequences may be required to induce a memory response that can be recalled during colonisation.

It is interesting to note that the Intimin epitopes identified in this study sit in a region of the molecule that is unlikely to be accessible to host antibodies. Mathematical modelling has identified signal peptides and transmembrane domains as molecule regions that are particularly dense in CD8^+^ T-cell epitopes [30]. This study suggests that this may also be the case for CD4^+^ T-cell epitopes. Whether this is due to their inherent hydrophobicity or part of a regulatory mechanism to ensure that B cell responses only receive T-cell help if they are directed against epitopes that originate from a full length pathogen molecule is unclear.

Antibodies raised to the EHEC O157:H7 recombinant C-terminal fragment Intimin-531 cross react with whole cell preparations from six different EHEC serotypes: O111, O103:H2, O145, O26:H11, O26:H- and O26 [31], despite the variability in the extra-cellular domains between the different Intimin types represented by these strains. Taken together, both the CD4^+^ T-cell epitopes identified in this study and antibody epitopes identified in previous studies suggest that immunisation with Intimin has the potential to provide cross-protection against different EHEC serotypes.

In conclusion, this study demonstrates that CD4^+^ T-cells from cattle colonised with EHEC O157:H7 recognise and respond to peptide sequences from 7/16 EHEC O157:H7 proteins tested. The most commonly recognised molecule was the N-terminal region of Intimin, highlighting important considerations with respect to the use of C-terminal Intimin fragments in EHEC O157:H7 vaccine development. The failure to identify CD4^+^ T-cell responses against H7 flagellin raises questions as to the mechanism of anti-flagellin antibody generation in cattle by EHEC O157:H7.

## Additional files

10.1186/s13567-016-0374-5 **Details of EHEC O157 protein sequences for epitope mapping.** Names and accession numbers of 16 EHEC O157 protein sequences used to synthesise peptides for T-cell epitope mapping.
10.1186/s13567-016-0374-5 **Pooling strategy for EHEC O157 peptides.** Details of peptide pools used to stimulate CD4^+^ T-cell lines from rectal lymph nodes of EHEC O157 challenged calves.
10.1186/s13567-016-0374-5 ***Theileria parva*** **peptide sequences.** Amino acid sequences of *T. parva* peptides used as negative controls for CD4^+^ T-cell stimulation assays.
10.1186/s13567-016-0374-5 **IFN-γ production by CD4** ^**+**^ **T-cells from calves 101398 and 101053 in response to pooled EHEC O157 peptides.** Graphs represent IFN-γ released from CD4^+^ T-cell lines generated from the rectal lymph nodes of calves 101398 and 101053 in response to EHEC O157 peptide stimulation. Peptide pools are ordered on the Y axis by the amount of IFN-γ produced. Solid lines denote the mean of two negative control wells. Dotted lines denote three standard deviations from the negative control.
10.1186/s13567-016-0374-5 **Sequence alignment of Intimin epitopes against Intimin sequences from non-O157 EHEC serotypes.** Alignment of Intimin CD4^+^ T-cell epitope sequences with representative Intimin sequences from EHEC serotypes O145, O127, O26, O103, O121, O45 and O111. Percentage values indicate % similarity to the EHEC O157:H7 reference sequence.
