# Supplementary figures and images for: Identification of epitopes recognised by mucosal CD4+ T-cell populations from cattle experimentally colonised with Escherichia coli O157:H7
Source: Vet Res. 2016 Sep 2;47(1):90. doi: 10.1186/s13567-016-0374-5 (PMC5010706; doi:10.1186/s13567-016-0374-5)

101398

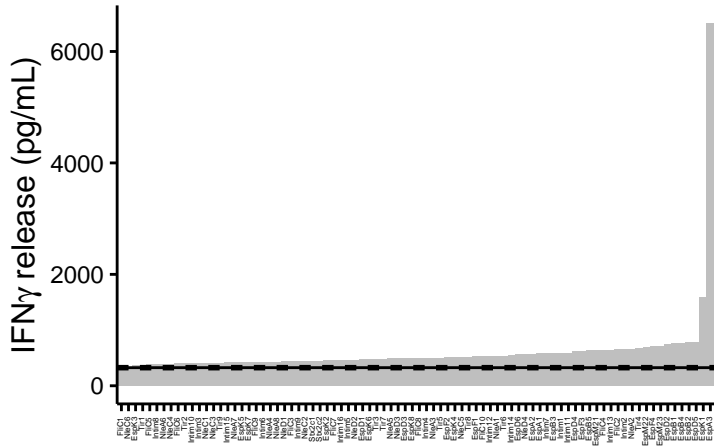

101053

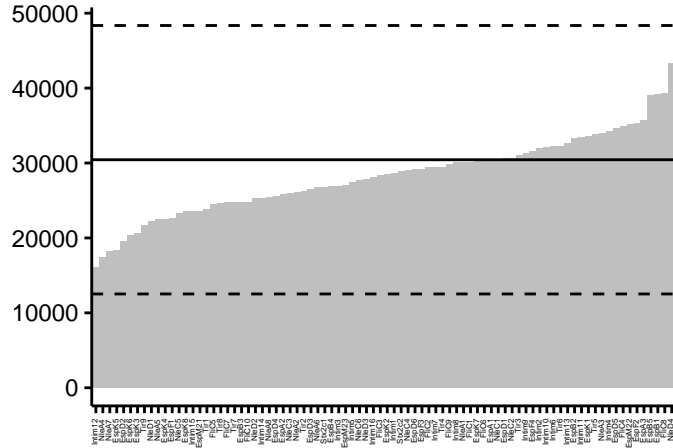

Supplement: Supplementary file 4 — 10.1186/s13567-016-0374-5 IFN-γ production by CD4 + T-cells from calves 101398 and 101053 in response to pooled EHEC O157 peptides. Graphs represent IFN-γ released from CD4+ T-cell lines generated from the rectal lymph nodes of calves 101398 and 101053 in response to EHEC O157 peptide stimulation. Peptide pools are ordered on the Y axis by the amount of IFN-γ produced. Solid lines denote the mean of two negative control wells. Dotted lines denote three standard deviations from the negative control. [file 13567_2016_374_MOESM4_ESM.pdf]
