# Supplementary material for: Identification of epitopes recognised by mucosal CD4+ T-cell populations from cattle experimentally colonised with Escherichia coli O157:H7
Source: Vet Res. 2016 Sep 2;47(1):90. doi: 10.1186/s13567-016-0374-5 (PMC5010706; doi:10.1186/s13567-016-0374-5)
Supplement: Supplementary file 5 — 10.1186/s13567-016-0374-5 Sequence alignment of Intimin epitopes against Intimin sequences from non-O157 EHEC serotypes. Alignment of Intimin CD4+ T-cell epitope sequences with representative Intimin sequences from EHEC serotypes O145, O127, O26, O103, O121, O45 and O111. Percentage values indicate % similarity to the EHEC O157:H7 reference sequence. [file 13567_2016_374_MOESM5_ESM.pdf]

```
Reference sequence (1): O157:H7.gamma
Identities normalised by aligned length.
Colored by: identity + property

                                   1 [        .         .         .         .         :         .         .         .         .         1         .         . 120
 1 O157:H7.gamma     100.0%   MITHGCYTRTRHKHKLKKTLIMLSAGLGLFFYVNQNSFANGENYFKLGSDSKLLTHDSYQNRLFYTLKTGETVADLSKSQDINLSTIWSLNKHLYSSESEMMKAAPGQQIILPLKKLPFE
 2 O145:H28.gamma     99.8%   MITHGCYTRTRHKHKLKKTLIMLSAGLGLFFYVNQNSFANGENYFKLGSDSKLLTHDSYQNRLFYTLKTGETVADLSKSQDINLSTIWSLNKHLYSSESEMMKAAPGQQIILPLKKLPFE
 3 O127:H6.alpha      82.6%   MITHGFYARTRHKHKLKKTFIMLSAGLGLFFYVNQNSFANGENYFKLGSDSKLLTHNSYQNRLFYTLKTGETVADLSKSQDINLSTIWSLNKHLYSSESEMMKAAPGQQIILPLKKLPFE
 4 O26:H11.beta       82.1%   MITHGFYARTRHKHKLKKTFIMLSAGLGLFFYVNQNSFANGENYFKLSSDSKLLTQNAAQDRLFYTLKTGETVANISKSQGISLSVIWSLNKHLYSSESEMMKAGPGQQIILPLKKLSVE
 5 O103:H2.epsilon    81.6%   MITHGFYTRTRHKHKLKKTFIMLSAGLGLFFYVNQNSFANGENYFKLSSDSKLLTQNAAQDRLFYTLKTGETVANISKSQGISLSVIWSLNKHLYSSESEMMKAGPGQQIILPLKKLSVE
 6 O121:H19.epsilon   81.6%   MITHGFYTRTRHKHKLKKTFIMLSAGLGLFFYVNQNSFANGENYFKLSSDSKLLTQNAAQDRLFYTLKTGETVANISKSQGISLSVIWSLNKHLYSSESEMMKAGPGQQIILPLKKLSVE
 7 O45:H2.epsilon     81.6%   MITHGFYTRTRHKHKLKKTFIMLSAGLGLFFYVNQNSFANGENYFKLSSDSKLLTQNAAQDRLFYTLKTGETVANISKSQGISLSVIWSLNKHLYSSESEMMKAGPGQQIILPLKKLSVE
 8 O111:H-.theta      88.8%   MITHGFYARTRHKHKLKKTFIMLSAGLGLFFYVNQNSFANGENYFKLSSDSKLLTQNVAQDRLFYTLKTGETVSSISKSQGISLSVIWSLNKHLYSSESEMLKAAPGQQIILPLKKLSVE
 9 Intim1.1          100.0%   MITHGCYTRTRHKHKLKK------------------------------------------------------------------------------------------------------
10 Intim1.4          100.0%   ------------------TLIMLSAGLGLFFYVNQN------------------------------------------------------------------------------------
11 Intim1.6          100.0%   ------------------------------FYVNQNSFANGENYFKLG------------------------------------------------------------------------
12 Intim1.10         100.0%   ------------------------------------------------------THDSYQNRLFYTLKTGET------------------------------------------------
13 Intim2.8          100.0%   ------------------------------------------------------------------------------------------------------KAAPGQQIILPLKKLPFE
14 Intim2.9          100.0%   ------------------------------------------------------------------------------------------------------------QIILPLKKLPFE
15 Intim7.7          100.0%   ------------------------------------------------------------------------------------------------------------------------

                               121        .         .         :         .         .         .         .         2         .         .         .         . 240
 1 O157:H7.gamma     100.0%   YSALPLLGSAPLVAAGGVAGHTNKLTKMSPDVTKSNMTDDKALNYAAQQAASLGSQLQSRSLNGDYAKDTALGIAGNQASSQLQAWLQHYGTAEVNLQSGNNFDGSSLDFLLPFYDSEKM
 2 O145:H28.gamma     99.8%   YSALPLLGSAPLVAAGGVAGHTNKLTKMSPDVTKSNMTDDKALNYAAQQAASLGSQLQSRSLNGDYAKDTALGIAGNQASSQLQAWLQHYGTAEVNLQSGNNFDGSSLDFLLPFYDSEKM
 3 O127:H6.alpha      82.6%   YSALPLLGSAPLVAAGGVAGHTNKLTKMSPDVTKSNMTDDKALNYAAQQAASLGSQLQSRSLNGDYAKDTALGIAGNQASSQLQAWLQHYGTAEVNLQSGNNFDGSSLDFLLPFYDSEKM
 4 O26:H11.beta       82.1%   YSALPVLGSAPVVAAGGVAGHTNKMTKMSPDATKSNTTDDKALNYAAQQAASLGSQLQSRSLNGDYAKDTALGMASSQASSQLQAWLQHYGTAEVNLQSGNNFDGSSLDFLLPFYDSENM
 5 O103:H2.epsilon    81.6%   YSALPVLGSAPVVAAGGVAGHTNKMTKMSPDATKSNTTDDKALNYAAQQAASLGSQLQSRSLNGDYAKDTALGMASSQASSQLQAWLQHYGTAEVNLQSGNNFDGSSLDFLLPFYDSENM
 6 O121:H19.epsilon   81.6%   YSALPVLGSAPVVAAGGVAGHTNKMTKMSPDATKSNTTDDKALNYAAQQAASLGSQLQSRSLNGDYAKDTALGMASSQASSQLQAWLQHYGTAEVNLQSGNNFDGSSLDFLLPFYDSENM
 7 O45:H2.epsilon     81.6%   YSALPVLGSAPVVAAGGVAGHTNKMTKMSPDATKSNTTDDKALNYAAQQAASLGSQLQSRSLNGDYAKDTALGMASSQASSQLQAWLQHYGTAEVNLQSGNNFDGSSLDFLLPFYDSENM
 8 O111:H-.theta      88.8%   YGALPVLGSAPVVAAGGVAGHTNKMTKMSPDATQSNMTDDKALNYTAQQAASLGSQLQSRSLHGDYAKDTALGIAGNQASSQLQAWLQHYGTAEVNLQSGNNFDGSSLDFLLPFYDSEKM
 9 Intim1.1          100.0%   ------------------------------------------------------------------------------------------------------------------------
10 Intim1.4          100.0%   ------------------------------------------------------------------------------------------------------------------------
11 Intim1.6          100.0%   ------------------------------------------------------------------------------------------------------------------------
12 Intim1.10         100.0%   ------------------------------------------------------------------------------------------------------------------------
13 Intim2.8          100.0%   ------------------------------------------------------------------------------------------------------------------------
14 Intim2.9          100.0%   YSALPL------------------------------------------------------------------------------------------------------------------
15 Intim7.7          100.0%   ------------------------------------------------------------------------------------------------------------------------

                               241        :         .         .         .         .         3         .         .         .         .         :         . 360
 1 O157:H7.gamma     100.0%   LAFGQVGARYIDSRFTANLGAGQRFFLPANMLGYNVFIDQDFSGDNTRLGIGGEYWRDYFKSSVNGYFRMSGWHESYNKKDYDERPANGFDIRFNGYLPSYPALGAKLIYEQYYGDNVAL
 2 O145:H28.gamma     99.8%   LAFGQVGARYIDSRFTANLGAGQRFFLPANMLGYNVFIDQDFSGDNTRLGIGGEYWRDYFKSSVNGYFRMSGWHESYNKKDYDERPANGFDIRFNGYLPSYPALGAKLIYEQYYGDNVAL
 3 O127:H6.alpha      82.6%   LAFGQVGARYIDSRFTANLGAGQRFFLPENMLGYNVFIDQDFSGDNTRLGIGGEYWRDYFKSSVNGYFRMSGWHESYNKKDYDERPANGFDIRFNGYLPSYPALGAKLMYEQYYGDNVAL
 4 O26:H11.beta       82.1%   LAFGQVGARYIDSRFTANLGAGQRFFLPENMLGYNVFIDQDFSGDNTRLGIGGEYWRDYFKSSVNGYFRMSGWHESYNKKDYDERPANGFDIRFNGYLPSYPALGAKLMYEQYYGDNVAL
 5 O103:H2.epsilon    81.6%   LAFGQVGARYIDSRFTANLGAGQRFFLPENMLGYNVFIDQDFSGDNTRLGIGGEYWRDYFKSSVNGYFRMSGWHESYNKKDYDERPANGFDIRFNGYLPSYPALGAKLMYEQYYGDNVAL
 6 O121:H19.epsilon   81.6%   LAFGQVGARYIDSRFTANLGAGQRFFLPENMLGYNVFIDQDFSGDNTRLGIGGEYWRDYFKSSVNGYFRMSGWHESYNKKDYDERPANGFDIRFNGYLPSYPALGAKLMYEQYYGDNVAL
 7 O45:H2.epsilon     81.6%   LAFGQVGARYIDSRFTANLGAGQRFFLPENMLGYNVFIDQDFSGDNTRLGIGGEYWRDYFKSSVNGYFRMSGWHESYNKKDYDERPANGFDIRFNGYLPSYPALGAKLMYEQYYGDNVAL
 8 O111:H-.theta      88.8%   LAFGQVGARYIDSRFTANLGAGQRFFLPENMLGYNVFIDQDFSGDNTRLGIGGEYWRDYFKSSVNGYFRMSGWHESYNKKDYDERPANGFDIRFNGYLPSYPALGAKLMYEQYYGDNVAL
 9 Intim1.1          100.0%   ------------------------------------------------------------------------------------------------------------------------
10 Intim1.4          100.0%   ------------------------------------------------------------------------------------------------------------------------
11 Intim1.6          100.0%   ------------------------------------------------------------------------------------------------------------------------
12 Intim1.10         100.0%   ------------------------------------------------------------------------------------------------------------------------
13 Intim2.8          100.0%   ------------------------------------------------------------------------------------------------------------------------
14 Intim2.9          100.0%   ------------------------------------------------------------------------------------------------------------------------
```

```
15 Intim7.7          100.0%   ------------------------------------------------------------------------------------------------------------------------

                          361            .             .             .             4             .             .             .             .             :             .             .             .   480
 1 O157:H7.gamma      100.0%   FNSDKLQSNPGAATVGVNYTPIPLVTMGIDYRHGTGNENDLLYSMQFRYQFDKSWSQQIEPQYVNELRTLSGSRYDLVQRNNNIILEYKKQDILSLNIPHDINGTEHSTQKIQLIVKSKY
 2 O145:H28.gamma      99.8%   FNSDKLQSNPGAATVGVNYTPIPLVTMGIDYRHGTGNENDLLYSMQFRYQFDKSWSQQIEPQYVNELRTLSGSRYDLVQRNNNIILEYKKQDILSLNIPHDINGTEHSTQKIQLIVKSKY
 3 O127:H6.alpha       82.6%   FNSDKLQSNPGAATVGVNYTPIPLVTMGIDYRHGTGNENDLLYSMQFRYQFDKPWSQQIEPQYVNELRTLSGSRYDLVQRNNNIILEYKKQDILSLNIPHDINGTERSTQKIQLIVKSKY
 4 O26:H11.beta        82.1%   FNSDKLQSNPGAATVGVNYTPIPLVTMGIDYRHGTGNENDLLYSMQFRYQFDKPWSQQIEPQYVNELRTLSGSRYDLVQRNNNIILEYKKQDILSLNIPHDINGTEHSTQKIQLIVKSKY
 5 O103:H2.epsilon     81.6%   FNSDKLQSNPGAATVGVNYTPIPLVTMGIDYRHGTGNENDLLYSMQFRYQFDKPWSQQIEPQYVNELRTLSGSRYDLVQRNNNIILEYKKQDILSLNIPHDINGTEHSTQKIQLIVKSKY
 6 O121:H19.epsilon    81.6%   FNSDKLQSNPGAATVGVNYTPIPLVTMGIDYRHGTGNENDLLYSMQFRYQFDKPWSQQIEPQYVNELRTLSGSRYDLVQRNNNIILEYKKQDILSLNIPHDINGTEHSTQKIQLIVKSKY
 7 O45:H2.epsilon      81.6%   FNSDKLQSNPGAATVGVNYTPIPLVTMGIDYRHGTGNENDLLYSMQFRYQFDKPWSQQIEPQYVNELRTLSGSRYDLVQRNNNIILEYKKQDILSLNIPHDINGTEHSTQKIQLIVKSKY
 8 O111:H-.theta       88.8%   FNSDKLQSNPGAATVGVNYTPIPLVTMGIDYRHGTGNENDLLYSMQLRYQFDKPWSQQIEPQYVNELRTLSGSRYDLVQRNNNIILEYKKQDILSLNIPHDINGTEHSTQKIQLIVKSKY
 9 Intim1.1          100.0%   ------------------------------------------------------------------------------------------------------------------------
10 Intim1.4          100.0%   ------------------------------------------------------------------------------------------------------------------------
11 Intim1.6          100.0%   ------------------------------------------------------------------------------------------------------------------------
12 Intim1.10         100.0%   ------------------------------------------------------------------------------------------------------------------------
13 Intim2.8          100.0%   ------------------------------------------------------------------------------------------------------------------------
14 Intim2.9          100.0%   ------------------------------------------------------------------------------------------------------------------------
15 Intim7.7          100.0%   ------------------------------------NENDLLYSMQFRYQFDKS------------------------------------------------------------------

                          481            .         5   .             .             .             .             :             .             .             .             .           6   600
 1 O157:H7.gamma      100.0%   GLDRIVWDDSALRSQGGQIQHSGSQSAQDYQAILPAYVQGGSNIYKVTARAYDRNGNSSNNVQLTITVLSNGQVVDQVGVTDFTADKTSAKADNADTITYTATVKKNGVAQANVPVSFNI
 2 O145:H28.gamma      99.8%   GLDRIVWDDSALRSQGGQIQHSGSQSAQDYQAILPAYVQGGSNIYKVTARAYDRNGNSSNNVQLTITVLSNGQVVDQVGVTDFTADKTSAKADNADTITYTATVKKNGVAQANVPVSFNI
 3 O127:H6.alpha       82.6%   GLDRIVWDDSALRSQGGQIQHSGSQSAQDYQAILPAYVQGGSNVYKVTARAYDRNGNSSNNVLLTITVLSNGQVVDQVGVTDFTADKTSAKADGTEAITYTATVKKNGVAQANVPVSFNI
 4 O26:H11.beta        82.1%   GLDRIVWDDSALRSQGGQIQHGGSQSAQDYQAILPAYVQGGSNIYKVTARAYDRNGNSSNNVQLTITVLPNGQVVDQVGVTDFTADKTSAKADGIEAITYTATVKKNGVAQANVPVTFSI
 5 O103:H2.epsilon     81.6%   GLDRIVWDDSALRSQGGQIQHSGSQSAQDYQAILPAYVQGGSNIYKVTARAYDRNGNSSNNVQLTITVLPNGQVVDQVGVTDFTADKTSAKADNVDTITYTATVKKNGVAQANAPVTFSI
 6 O121:H19.epsilon    81.6%   GLDRIVWDDSALRSQGGQIQHSGSQSAQDYQAILPAYVQGGSNIYKVTARAYDRNGNSSNNVQLTITVLPNGQVVDQVGVTDFTADKTSAKADNVDTITYTATVKKNGVAQANAPVTFSI
 7 O45:H2.epsilon      81.6%   GLDRIVWDDSALRSQGGQIQHSGSQSAQDYQAILPAYVQGGSNIYKVTARAYDRNGNSSNNVQLTITVLPNGQVVDQVGVTDFTADKTSAKADNVDTITYTATVKKNGVAQANAPVTFSI
 8 O111:H-.theta       88.8%   GLDRIVWDDSALRSQGGQIQHSGSQSAQDYQAILPAYVQGGSNIYKVTARAYDRNGNSSNNVQLTITVLSNGQVVDQVGVTDFTADKTSAKADGTEAITYTATVKKNGVTQANVPVSFNI
 9 Intim1.1          100.0%   ------------------------------------------------------------------------------------------------------------------------
10 Intim1.4          100.0%   ------------------------------------------------------------------------------------------------------------------------
11 Intim1.6          100.0%   ------------------------------------------------------------------------------------------------------------------------
12 Intim1.10         100.0%   ------------------------------------------------------------------------------------------------------------------------
13 Intim2.8          100.0%   ------------------------------------------------------------------------------------------------------------------------
14 Intim2.9          100.0%   ------------------------------------------------------------------------------------------------------------------------
15 Intim7.7          100.0%   ------------------------------------------------------------------------------------------------------------------------

                          601            .             .             .             .             :             .             .             .             .             7             .             .   720
 1 O157:H7.gamma      100.0%   VSGTATLGANSAKTDANGKATVTLKSSTPGQVVVSAKTAEMTSALNASAVIFFDQTKASITEIKADKTTAVANGKDAIKYTVKVMKNGQPVNNQSVTFSTNFGMFNGKSQTQA-TTGNDG
 2 O145:H28.gamma      99.8%   VSGTATLGANSAKTDANGKATVTLKSSTPGQVVVSAKTAEMTSALNASAVIFFDQTKASITEIKADKTTAVANGKDAIKYTVKVMKNGQPVNNQSVTFSTNFGMFNGKSQTQA-TTGNDG
 3 O127:H6.alpha       82.6%   VSGTAVLSANSANTNGSGKATVTLKSDKPGQVVVSAKTAEMTSALNANAVIFVDQTKASITEIKADKTTAVANGQDAITYTVKVMKGDKPVSNQEVTFTTTLGKLSNS----TEKTDTNG
 4 O26:H11.beta        82.1%   VSGTATLGANSARTDGNGKATVTLKSATPGQVVVSAKTAEMTSPLNASAVIFVDQTKASITEIKADKTTAKADGSDAITYTVRVMKEGAPVVDQKVTFSKDFGTLNKT---EA-TTDQNG
 5 O103:H2.epsilon     81.6%   VSGTATLGANSAKTDGNGKATVTLKSGTPGQVVVSAKTAEMTSPLNASAVIFVDQTKASITEIKADKTTAKANGSDAITYIVKVMKNNQPEANHSVTFSTNFGNLGGNSNTQIVKTDKDG
 6 O121:H19.epsilon    81.6%   VSGTATLGANSAKTDGNGKATVTLKSGTPGQVVVSAKTAEMTSPLNASAVIFVDQTKASITEIKADKTTAKANGSDAITYIVKVMKNNQPEANHSVTFSTNFGNLGGNSNTQIVKTDKDG
 7 O45:H2.epsilon      81.6%   VSGTATLGANSAKTDGNGKATVTLKSGTPGQVVVSAKTAEMTSPLNASAVIFVDQTKASITEIKADKTTAKANGSDAITYIVKVMKNNQPEANHSVTFSTNFGNLGGNSNTQIVKTDKDG
 8 O111:H-.theta       88.8%   VSGTATLGANSATTDANGKATVTLKSSTPGQVVVSAKTAEMTSALNASAVIFVEQTKASITEIKADKTTAVANGNDAVTYTVKVMKEGQPVQGHSVAFTTNFGMFNGKSQTQNATTGSDG
 9 Intim1.1          100.0%   ------------------------------------------------------------------------------------------------------------------------
10 Intim1.4          100.0%   ------------------------------------------------------------------------------------------------------------------------
11 Intim1.6          100.0%   ------------------------------------------------------------------------------------------------------------------------
12 Intim1.10         100.0%   ------------------------------------------------------------------------------------------------------------------------
13 Intim2.8          100.0%   ------------------------------------------------------------------------------------------------------------------------
14 Intim2.9          100.0%   ------------------------------------------------------------------------------------------------------------------------
15 Intim7.7          100.0%   ------------------------------------------------------------------------------------------------------------------------

                          721            .             .             :             .             .             .             .             8             .             .             .             .   840
```

```
 1 O157:H7.gamma    100.0%    RATITLTSSSAGKATVSATVS-DGAEVKATEVTFFDELKIDN-KVDIIGNNVRGELPNIWLQYGQFKLKASGGDGTYSWYSENTSIATVDA-SGKVTLNGKGSVVIKATSGDKQTVSYTI
 2 O145:H28.gamma    99.8%    RATITLTSSSTGKATVSATVS-DGAEVKATEVTFFDELKIDN-KVDIIGNNVRGELPNIWLQYGQFKLKASGGDGTYSWYSENTSIATVDA-SGKVTLNGKGSVVIKATSGDKQTVSYTI
 3 O127:H6.alpha     82.6%    YAKVTLTSTTPGKSLVSARVSDVAVDVKAPEVEFFTTLTIDDGNIEIVGTGVKGKLPTVWLQYGQVNLKASGGNGKYTWRSANPAIASVDASSGQVTLKEKGTTTISVISSDNQTATYTI
 4 O26:H11.beta      82.1%    YATVKLSSNTPGKAIVSAKVSGVGTEVKATTVEFFAPLSIDGDKVTVIGTGITGALPKNWLQYGQVKLQATGGNGKYTWKSSNTKIASVDN-SGVITLNEKGSATITVVSGDNQSATYTI
 5 O103:H2.epsilon   81.6%    RATVKLTSGVAGNAVVSAKVSEVNTEVKAPEVKFFSVLSIDS-NVSIIGTSANGALPNIWLRYGQFKLTAKGGDGKYQWRSQDPSVASVDALTGRVTLLKKGTTTIEVVSGDNQTAMYTI
 6 O121:H19.epsilon  81.6%    RATVKLTSGVAGNAVVSAKVSEVNTEVKAPEVKFFSVLSIDS-NVSIIGTSANGALPNIWLRYGQFKLTAKGGDGKYQWRSQDPSVASVDALTGRVTLLKKGTTTIEVVSGDNQTAMYTI
 7 O45:H2.epsilon    81.6%    RATVKLTSGVAGNAVVSAKVSEVNTEVKAPEVKFFSVLSIDS-NVSIIGTSANGALPNIWLRYGQFKLTAKGGDGKYQWRSQDPSVASVDALTGRVTLLKKGTTTIEVVSGDNQTAMYTI
 8 O111:H-.theta     88.8%    RATITLTSSSAGKATVSATVSG-GNDVKAPEVTFFDGLKIDN-KVDILGKNVTGDLPNIWLQYGQFKLKVSGGNGTYSWHSENTNIATVDE-SGKVTLKGKGTAVINVTSGDKQTVSYTI
 9 Intim1.1         100.0%    ------------------------------------------------------------------------------------------------------------------------
10 Intim1.4         100.0%    ------------------------------------------------------------------------------------------------------------------------
11 Intim1.6         100.0%    ------------------------------------------------------------------------------------------------------------------------
12 Intim1.10        100.0%    ------------------------------------------------------------------------------------------------------------------------
13 Intim2.8         100.0%    ------------------------------------------------------------------------------------------------------------------------
14 Intim2.9         100.0%    ------------------------------------------------------------------------------------------------------------------------
15 Intim7.7         100.0%    ------------------------------------------------------------------------------------------------------------------------

                        841          :         .         .         .         .    9         .         .         .         .   ] 950
 1 O157:H7.gamma    100.0%    KAPSYMIK-VD--KQAYYADAMSICKN---LLPSTQTVLSDIYDSWGAANKYSHYSSMNSITAWIKQTSSEQRSGVSSTYNLITQNPLPGV------NVNTPNVYAVCVE
 2 O145:H28.gamma    99.8%    KAPSYMIK-VD--KQAYYADAMSICKN---LLPSTQTVLSDIYDSWGAANKYSHYSSMNSITAWIKQTSSEQSSGVSSTYNLITQNPLPGV------NVNTPNVYAVCVE
 3 O127:H6.alpha     82.6%    ATPNSLIV-PNMSKRVTYNDAVNTCKNFGGKLPSSQNELENVFKAWGAANKYEYYKSSQTIISWVQQTAQDAKSGVASTYDLVKQNPLNNI------KASESNAYATCVK
 4 O26:H11.beta      82.1%    NAPGSIVIAVDKNTRVTYFDAENKCKTNSANLAQSKELLANIYSTWGAANKYPYYSGSKSLTAWIKQSSSEQSSGVSSTYDLVTKNQLINV------GVNNKNAFSVCVK
 5 O103:H2.epsilon   81.6%    NTPTKFIS-VETQNKVVYSDAEATCRMNNARLPSSTSELKDVYNKWGAANSYEGYKGKKTITAWTQQTEDDKQKGWTSTFDIVTKNEIPSNGSNSKVHVNKANAFAVCVR
 6 O121:H19.epsilon  81.6%    NTPTKFIS-VETQNKVVYSDAEATCRMNNARLPSSTSELKDVYNKWGAANSYEGYKGKKTITAWTQQTEDDKQKGWTSTFDIVTKNEIPSNGSNSKVHVNKANAFAVCVR
 7 O45:H2.epsilon    81.6%    NTPTKFIS-VETQNKVVYSDAEATCRMNNARLPSSTSELKDVYNKWGAANSYEGYKGKKTITAWTQQTEDDKQKGWTSTFDIVTKNEIPSNGSNSKVHVNKANAFAVCVR
 8 O111:H-.theta     88.8%    KAPNYMIRV---GNKASYANAMSFCGN---LLPSSQTVLSNVYNSWGPANGYDHYRSMQSITAWITQTEADKISGVSTTYDLITQNPHKDV------TLNAPNVYAVCVE
 9 Intim1.1         100.0%    -----------------------------------------------------------------------------------------------------------
10 Intim1.4         100.0%    -----------------------------------------------------------------------------------------------------------
11 Intim1.6         100.0%    -----------------------------------------------------------------------------------------------------------
12 Intim1.10        100.0%    -----------------------------------------------------------------------------------------------------------
13 Intim2.8         100.0%    -----------------------------------------------------------------------------------------------------------
14 Intim2.9         100.0%    -----------------------------------------------------------------------------------------------------------
15 Intim7.7         100.0%    -----------------------------------------------------------------------------------------------------------
```
